# Metabolomics and Cheminformatics Analysis of Antifungal Function of Plant Metabolites

**DOI:** 10.3390/metabo6040031

**Published:** 2016-09-30

**Authors:** Miroslava Cuperlovic-Culf, NandhaKishore Rajagopalan, Dan Tulpan, Michele C. Loewen

**Affiliations:** National Research Council of Canada, 1200 Montreal Road, Ottawa, ON K1A 0R6, Canada; NandhaKishore.Rajagopalan@nrc-cnrc.gc.ca (N.R.); Dan.Tulpan@nrc-cnrc.gc.ca (D.T.); Michele.Loewen@nrc-cnrc.gc.ca (M.C.L.)

**Keywords:** Fusarium head blight, *Fusarium graminearum*, cheminformatics, metabolomics, plant resistance, biotic stress, antifungal, carbonic anhydrase, cytochrome P450

## Abstract

Fusarium head blight (FHB), primarily caused by *Fusarium graminearum*, is a devastating disease of wheat. Partial resistance to FHB of several wheat cultivars includes specific metabolic responses to inoculation. Previously published studies have determined major metabolic changes induced by pathogens in resistant and susceptible plants. Functionality of the majority of these metabolites in resistance remains unknown. In this work we have made a compilation of all metabolites determined as selectively accumulated following FHB inoculation in resistant plants. Characteristics, as well as possible functions and targets of these metabolites, are investigated using cheminformatics approaches with focus on the likelihood of these metabolites acting as drug-like molecules against fungal pathogens. Results of computational analyses of binding properties of several representative metabolites to homology models of fungal proteins are presented. Theoretical analysis highlights the possibility for strong inhibitory activity of several metabolites against some major proteins in *Fusarium graminearum*, such as carbonic anhydrases and cytochrome P450s. Activity of several of these compounds has been experimentally confirmed in fungal growth inhibition assays. Analysis of anti-fungal properties of plant metabolites can lead to the development of more resistant wheat varieties while showing novel application of cheminformatics approaches in the analysis of plant/pathogen interactions.

## 1. Introduction

*Fusarium graminearum* is the primary pathogen causing Fusarium head blight (FHB) or scab, a devastating disease primarily affecting wheat and barley crops in humid and semi-humid areas worldwide. FHB causes major crop losses, as well as indirect losses due to grain contamination caused by potent mycotoxins, especially deoxyvalenol (DON) [1]. Strategies for the eradication of FHB include fungicide applications, cultural practices, and the longer-term development of genetic resistance in the plant. Although there are several registered fungicides for application in wheat and barley, none are very effective neither in the reduction of yield loss nor in the reduction of mycotoxin contamination [1,2,3]. Crops with genetic resistance have the highest potential and are a major breeding objective worldwide. DNA marker analysis has provided identification of quantitative trait loci (QTL) that provide partial resistance to some biotic stressors, including FHB [4]. Over one hundred FHB resistance-associated QTLs have been identified in wheat, but the specific functions of these QTLs is still largely unknown [4,5]. Genetic control of resistance can induce biochemical profile change leading to the resistance response. Biochemical resistance is directly associated with specific proteins and metabolites. Metabolomics methods have been used in a number of studies to determine major metabolic response induced by FHB infection in resistant and non-resistant wheat and barley, showing significant concentration changes in hormones, as well as primary and secondary metabolites, as a response to FHB. Analysis of metabolic factors affecting resistance is primarily aimed at detection of major effects of gene control on metabolism and possible new foci for further gene manipulation. Metabolomics data can also be used for the determination of biomarkers of resistance, infection, and plant response. Finally, metabolomics provides information about the resistance-related metabolites that can induce development of bio-inspired fungicides or help determine fungal protein targets for the development of specifically-targeted fungicides.

A number of secondary metabolites, including hormones, phenolic, and polyphenolic compounds, are significant in plant response to fungal infections. Hormones are metabolites that can easily circulate through parts of, or through the whole organism and that, even at low concentrations, signal and control responses, growth, and the development of organisms [6]. Plant hormones, including auxins, cytokinins (CK), gibberellins (GA), abscisic acid (ABA), ethylene (ET), bassino-steroids (BR), jasmonic acid (JA), and salicylic acid (SA), have major roles in plant defense against biotic and abiotic stressors [6,7]. Several functions of SA, JA, and ET have been described in great detail [8,9]. Other hormones, including ABA, auxin, GA, CK, BR, and strigolactones, have been implicated as important components of plant defense; however, their specific roles are not yet fully described. SA plays a major role in plant defense and is generally involved in the activation of defense responses against biotrophic and hemi-biotrophic pathogens where SA levels increase in pathogen challenged plants. Exogeneous applications of SA have been shown to induce expression of pathogenesis related genes (PR) [8,10,11,12]. JA and ET have stronger association with the defense against necrotrophic pathogens and herbivorous insects [13,14,15]. Auxin and ABA are also emerging as hormones involved in the regulation of the response to both biotrophic and necrotropic pathogens, although the mechanisms of their action are still not clear [7,16,17,18,19]. GA hormone is produced by plants, fungi, as well as bacteria, and appears to also have a significant role in plant disease progression, but the specific function has not been described [20].

The roles of phenolic compounds (including phytoalexins) in fungal responses of plants have been reviewed by Lattanzio et al. [21]. Phenolic compounds refer to all secondary natural metabolites arising from the shikimate-phenylpropanoids-flavonoids pathways. Antifungal phenolics, which include tannins and proanthocyanidins, can be either preformed (phytoanticipins) and redistributed following infections, or can be synthesized following interaction between the host and fungal parasite (phytoalexins). Over the last several years a number of metabolomics studies have been performed in order to determine plant metabolites associated with fungal infection, including FHB. Mass spectrometry was used for study of metabolic changes following FHB infection in resistant barley genotypes [22,23] as well as resistant wheat cultivars [5,24,25]. Nuclear magnetic resonance-based metabolite profiling was utilized for screening of passive resistance in wheat against FHB [26]. Finally, several volatile organic compounds produced by chickpeas show strong anti-FHB activity [27]. The effect of a small number of these metabolites has been tested in vitro. Phenoloic acids, including ferulic acid, are the most abundant in wheat bran. Following infection, phenolic acids are over-concentrated in resistant wheat and they have been demonstrated as efficient inhibitors of mycotoxin production in several strains of *Fusarium* [28,29]. Antifungal and antibacterial activity of tannic acid has been known for a long time [30,31,32], with activity against *Fusarium graminearum* shown recently [33].

The goal of the work presented here was to determine whether plant-produced metabolites can act as drug-like agents of the plant’s biotic response, and what are their possible protein targets. Cheminformatics analysis was used to identify plant metabolites with the highest potential to function as fungal growth inhibitors and, subsequently, the effect of several selected metabolites was tested experimentally. We present here a compilation of all previously determined resistance-related metabolites, followed by cheminformatics analysis of physicochemical properties and the relationships between these compounds. Possible protein targets of some of these compounds have been predicted using publicly available data for these, and related, molecules. The interaction potential between plant metabolites and proteins in fungus is explored computationally using high throughput small molecule docking. Subsequently, selected metabolites have been tested experimentally showing activity against *Fusarium graminearum* in fungal growth inhibition assays.

## 2. Results and Discussion

### 2.1. Selection of Metabolites and Cheminformatics Analysis of Their Molecular and Drug-Like Properties

**Figure 1:**
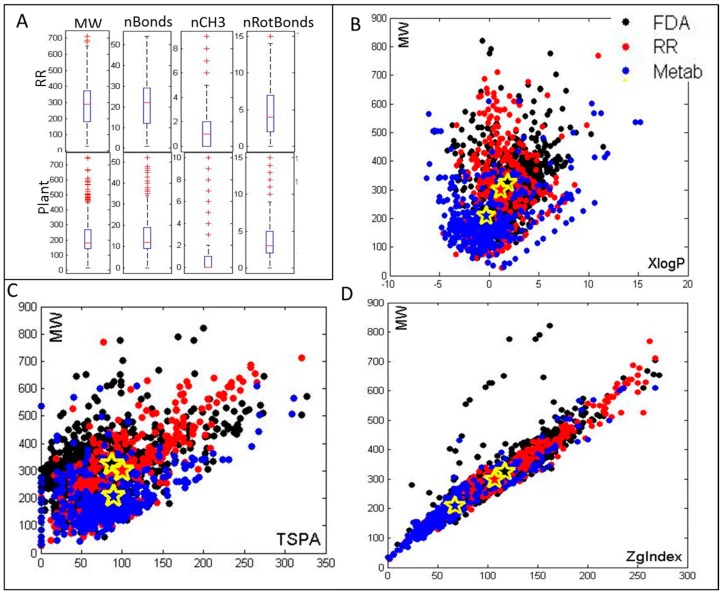
(**A**) Range of molecular sizes and structures in resistance-related (RR) metabolites, including the molecular weight (MW), the number of bonds (nBonds), the number of CH3 groups (nCH3), and the number of rotatable bonds (nRotBonds); (**B**–**D**) show values for Lipinski “rule-of-five” characteristics [42] of molecules in RR, plant metabolites (Metab), and FDA-approved drug (FDA) sets; (**B**) shows values of the logarithm of the partition coefficient for n-octanol/water LogP (XLogP) relative to MW; (**C**) topological polar surface area (TPSA) of molecules relative to MW and (**D**) Zagreb Index values (ZgIndex) relative to MW. Stars in (**B**–**D**) show the average values for all molecules in the group with black indicating FDA, red indicating RR, and blue indicating Metab. The larger similarity between RR and FDA molecules is apparent in all cases suggesting that RR metabolites have increased drug-like qualities, particularly in terms of passive membrane transport relative to average plant metabolite.

Metabolic changes resulting from FHB infection in resistant or less susceptible plants have been explored in wheat [5,24,25,32,33,34] and barley [23,35]. Anti-fusarium metabolites have also been explored in chickpeas, and their effect was tested for protection of wheat from FHB [27]. Additionally, investigations of metabolites commonly involved in biotic responses [21,36], as well as plant hormones with possible roles in resistance [7,18,37] have also been performed. Although there is some overlap in metabolites that accumulate as a response to biotic stress across these distinct experiments and across different species, there are also many unique metabolites identified in the different reports. Discrepancies in observed metabolic profiles can be a result of different behaviors of plants over time, in distinct conditions, or in different tissues. Metabolic coverage also depends on experimental procedures. Finally, continual improvements in sensitivity of experimental and analytical methods can introduce new metabolites to the measurements. In this work we have made a compilation of all metabolites determined in previously published studies dealing with FHB infection in grains. A complete list of these resistance related (RR) metabolites is provided in Supplementary Table S1. In total, 478 RR metabolites have been included in our database. Of 478 RR metabolites 474 are registered within PubChem [38] and, thus, have accessible structural information. Supplementary Table S1 includes PubChem [38] CIDs for these 474 metabolites, as well as compounds’ names provided in original references or in PubChem [38] for all RR metabolites. For metabolites that have not been registered in PubChem [38] Supplementary Table S1 shows IDs for either KNApSAcK [35], HMDB [39], and ChEBI [40] databases. These molecules belong to many different groups, including phenols, polyphenols, flavonoids, etc. Molecules can be classified based on their chemical characteristics, functional groups, size, biological pathways of origin, etc., and for many of the metabolites presented these types of classification are available in the original literature. Figure 1A shows the calculated values of several significant molecular characteristics for RR metabolites compared to those for all known plant metabolites (from PubChem [38] and Golm databases [41]), such as the molecular weight (MW), the number of both hydrogen acceptors and donors (HBA and HDA), and the number of heavy atoms (HA).

In order for RR metabolites to act as plant-produced antifungal agents they must have drug-like properties that can be simply represented through Lipinski “Rule-of-five” characteristics [42,43]. Lipinski rule-of-five is a standard method in drug discovery and its utility in antifungal development has been recently shown [43]. A comparison of Lipinski rules between RR metabolites, all known plant metabolites, and FDA approved drugs (Figure 1B–D) allows a quick investigation of the possibility for plant selection of drug-like characteristics in RR metabolites. A number of other molecular characteristics calculated for this set (in total 251 properties calculated using RCDK) including physical, chemical, and electronic properties again show similar ranges in the two groups of metabolites (data available from the authors). RR metabolites span a wide range of molecules from the highest molecular weight metabolites—tannic acid (MW = 1701.198; included as a general resistance related metabolite [21]) and Phyllanthusmin B (MW = 926.6506; produced by resistant wheat)—to the lowest molecular weight molecule, ethylene (general plant hormone). The size and number of hydrogen bond donors and acceptors for RR metabolites are for the large majority of RR metabolites within the Lipinski rule-of-five for drug-like molecules (Figure 1A).

The drug-like characteristics of RR metabolites are explored in comparison to all known plant metabolites in the Golm database [41] and all FDA approved drugs included in the ZINC database [44]. For this analysis we selected from PubChem [38] all RR metabolites that have available 3D structures, as well as any plant metabolites provided by the Golm database [41] with 3D structures. FDA-approved drugs with 3D structures from ZINC drug database were also selected [44]. This yielded 416 RR metabolites, 1171 plant metabolites, and 1985 FDA-drugs. For these metabolites we have calculated several physicochemical properties, including octanol/water partition coefficient (logP), molecular weight (MW), topological polar surface area (TPSA), and Zagreb Index using the RCDK package in R [45] (RCDK was developed by R. Guha), and comparisons between the RR metabolites, plant metabolites (Metab), and FDA drugs are shown in Figure 1B–D. Plots show (as filled yellow stars) average values for each group of metabolites. Although characteristics of molecules in all three groups are comparable, average MW:logP, MW:TPSA, and MW:Zagreb Index for RR and FDA molecules are more closely related, suggesting that RR metabolites generally have more drug-like characteristics than general plant metabolites.

The majority of RR metabolites have a larger average partition-coefficient (XlogP) value than what is seen in the overall plant metabolite set indicating their higher lipophilicity. XlogP is an atom-additive method for calculating the octanol/water partition coefficient (logP). It obtains the logP value for a compound by summing the contributions from component atoms and correction factors. Lipinski’s rule-of-five allowed range for XlogP is −0.4 to 5 and the majority of RR metabolites fit within these limits (Figure 1B). The topological polar surface area (TPSA) of molecules defines the sum of surfaces of polar atoms in a molecule (calculated using the method developed by [46]). In the currently used form, molecules with TPSA larger than 140 Å^2^ are believed to have a low capacity for unaided penetration through cell membranes, while those with TPSA less than 60 Å^2^ are easily absorbed by the cellular membrane [47]. Molecules with TPSA over 60 Å^2^ are believed to be favored and further regulated by molecular transporters, such as ABC transporters [48]. The average TSPA of RR metabolites is larger than for overall plant metabolites and highly comparable to the average for FDA-approved drugs (Figure 1C). It should be kept in mind that ABC transporters are crucial pathogen-related proteins highly overexpressed and involved in FHB resistance [49], possibly suggesting their role in active transport of RR metabolites. Figure 1D compares values for the Zagreb Index between RR, FDA, and plant metabolites. The Zagreb Index, is a topological measure of molecular branching, calculated as the sum of the squared vertex valences, i.e., the number of connections to heavy atoms regardless of their bond order [50]. Overall, the Zagreb Index values (ZgIndex) are significantly higher for RR than plant metabolites, and highly similar between RR and FDA molecules, although values for all three groups are still within the previously proposed, very large, optimal range of 22–452 [51].

### 2.2. Metabolite Activity

**Figure 2:**
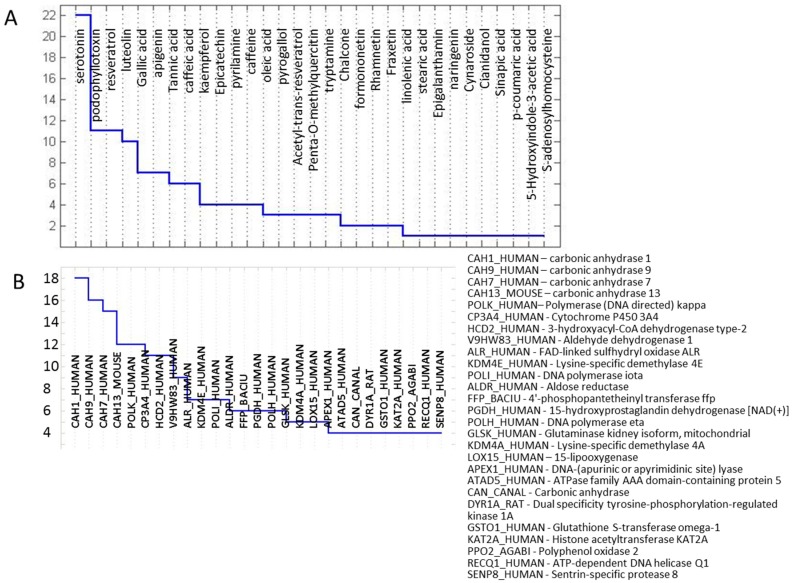
Known protein targets of RR metabolites. Shown are high-activity relationships where (**A**) shows metabolites with known activity against known protein targets; and (**B**) protein targets listed with more than four active metabolites determined from the subset of metabolites measured within the RR set. Protein names correspond to the UniProt database [52] names and are listed in the figure legend.

A number of biological assays have already been performed for many of the metabolites within the RR group. Protein targets are also known for many of these and this data is available through the PubChem database [38]. This information can guide us in proposing possible functions of tested, as well as related, molecules in the biotic response of a plant. Known activities for RR metabolites are shown in Figure 2. According to the publicly available tests, serotonin is the most promiscuous compound, showing binding with the largest number of tested proteins. The largest number of tested compounds target carbonic anhydrase proteins.

The RR metabolites show activity against a number of proteins and the complete list can be obtained from the authors. A number of metabolites have more than one known protein target and for several protein targets more than one metabolite shows activity (Figure 2).

A number of active metabolites listed in Figure 2 have been previously shown as antifungals. General antifungal and antibacterial activity of tannic acid, for example, has been known for a long time [30,31,32] and activity against *Fusarium graminearum* was recently experimentally shown [34]. Our analysis presents several possible protein targets for tannic acid, including carbonic anhydrases and a bromodomain adjacent to the zinc finger domain 2B, both having homolog in the *Fusarium graminearum* sequence. Many other targets of tannic acid have been defined in the literature. While the activity of tannic acid against *Fusarium gaminearum* has already been reported, it serves as a good positive control. At the same time, tannic acid is a large phenolic compound that has structural similarity to many other, simpler phenolic acids, detected in grains infected with FHB. Examples of these include ferulic acid, *p*-coumaric acid, salicylic acid, gallic acid, caffeic acid, etc. These significantly smaller products of the phenylpropanoid biosynthetic pathway possibly have similar protein binding targets as tannic acid.

Other highly active, tested metabolites include luteolin and serotonin, both structurally related to a number of other RR metabolites. Although their activities in bioassays have been extensively studied, their effect against *Fusarium graminearum* has, to the best of our knowledge, not been explored. Luteolin, as well as several other flavones with different hydroxylation patterns, have been previously identified as RR molecules with some protein targets already identified for apigenin and kaempferol (Figure 2). These, as well as several other luteolin-related molecules, have been observed in infected wheat [5,23]. Luteolin is a highly potent antioxidant and radical scavenger, similarly to other flavonoids. Likewise to other metabolites, the activity of luteolin has been primarily tested against mammalian proteins. Once again, many of the proteins that luteolin is active against have close protein sequence homologs in *Fusarium graminearum*. It is also interesting that glycosylated luteolin gets actively transported by ABC-type transporters [53]. Luteolin is structurally related to a number of other flavones and flavone derivatives within our RR metabolites set (e.g., isovitexin-7-O-xyloside, isovitexin 2″-O-(6′′′-feruloyl)glucoside, isovitexin-7-O-glucosyl-2″O-rhamnoside, and kaempferol-3-glucoside-7-rhamnoside, to name just a few).

Serotonin (5-hydroxytryptamine) is an extensively studied neurotransmitter in mammals, as well as a widely-distributed metabolite in plants. Serotonin is highly structurally similar to auxin (indol-3-acetic acid), one of the major plant hormones. It has been hypothesized that serotonin acts as an auxin function inhibitor, thereby regulating root development [54]. In resistant wheat infected with *Fusarium graminearum* a concentration increase has been observed in hydroxycinnamic acid amide-conjugated putrescine, tyramine, agmantine, and serotonin acting as pytoalexins [5]. Serotonin and its derivatives *p*-coumarylserotonin and feruloylserotonin were accumulated in *Bipolaris oryza* infected rice [55] where serotonin treatment suppresses the growth of fungal hyphae. Several other metabolites within the RR group also contain an indole ring and are highly similar to serotonin.

### 2.3. Protein Targets

**Figure 3:**
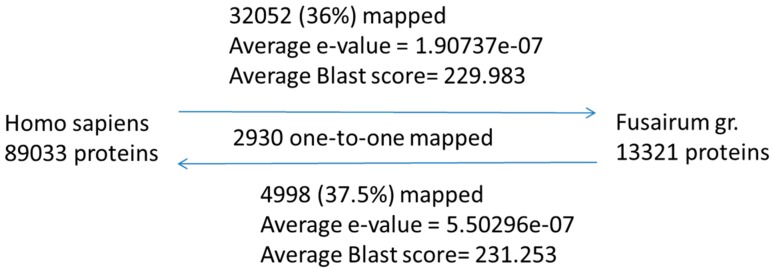
Statistical information regarding the mapping of human proteins to the *Fusarium graminearum* proteome. Sequence similarity was determined using in-house methodology.

As previous testing of biological activity of compounds was primarily performed on mammalian proteins, we have utilized sequence similarity studies and homology modelling to establish protein functions in wheat and *Fusarium graminearum* systems. Out of 327 protein targets obtained as possible action sites for the tested RR metabolites, 211 are *Homo sapiens* proteins. In order to determine relevance of this finding to interactions between plants and *Fusarium graminearum* we have mapped the complete human proteome against protein sequences available for *Fusarium graminearum* (strain PH-1; data obtained from the Broad Institute). Statistical information for complete human to complete *Fusarium graminearum* proteome mapping is shown in Figure 3. Out of all 89,033 human proteins obtained from UniProt database [52], 36% could be mapped to the 13,321 *Fusarium graminearum* proteins obtained from the Broad Institute database [56].

For the subset of 211 human proteins shown as targets of tested plant metabolites, 138 have significant homologs in the *Fusarium graminearum* sequence. Therefore, within the subset of metabolite-targeted proteins, 65% have homologs in *Fusarium graminearum*.

#### Homology Modelling and Docking Analysis—Activity of Metabolites against Fungal Targets

The majority of proteins listed as targets in Figure 2 have highly similar *Fusarium graminearum* protein homologs based on the sequence comparison performed in this work. For example, CA *Fusarium graminearum* sequence FGSG_04603 has the alpha CA superfamily domain and closest similarity to human CA (BLAST score: 7 × 10^−20^). Carbonic anhydrase (CA) is a known target of tannic acid [57], serotonin, ferulic acid [58], as well as number of other RR metabolites either through specific or non-specific binding and inhibition. At the same time CA has been indicated as an interesting target for antifungal agents (recently reviewed in [59]). Although several indicated metabolites have experimentally shown binding to human CA, binding to fusarium CA was not tested for any of the RR metabolites. In order to explore the possible binding of RR metabolites to fusarium CA we have performed high-throughput computational docking analysis.

For docking *Fusarium graminearum* CA was modelled using the homology modelling method provided by Swiss-Model [60,61,62], in comparison to proteins with highest sequence similarity with available X-ray 3D structures. *Fusarium graminearum* sequence FGSG_04603 was used as it was the closest homolog to several isozymes of human carbonic anhydrases of known structure.

A number of X-ray crystal structures are available for CA proteins, including representatives of different classes as well as distinct isozymes from human cells. Several available crystal structures include inhibitory molecules. Docking analysis for all RR metabolites was performed for human CA for comparison and *Fusarium graminearum* CA. Human CA I in complex with the inhibitor topiramate (pdb: 3LXE) was selected for the analysis of human protein. This is the structure of one of the known target proteins of serotonin and contains an inhibitor, allowing the determination of the grid zone for docking analysis. Fungal CA homolog FGST_04603 was modeled using crystal structure pdb: 3Q31 of the fungal alpha carbonic anhydrase from *Aspergillus oryzae*. Carbonic anhydrase from *Aspergillus oryzae* showed 39.32% sequence identity with *Fusarium graminearum* CA, studied here.

**Figure 4:**
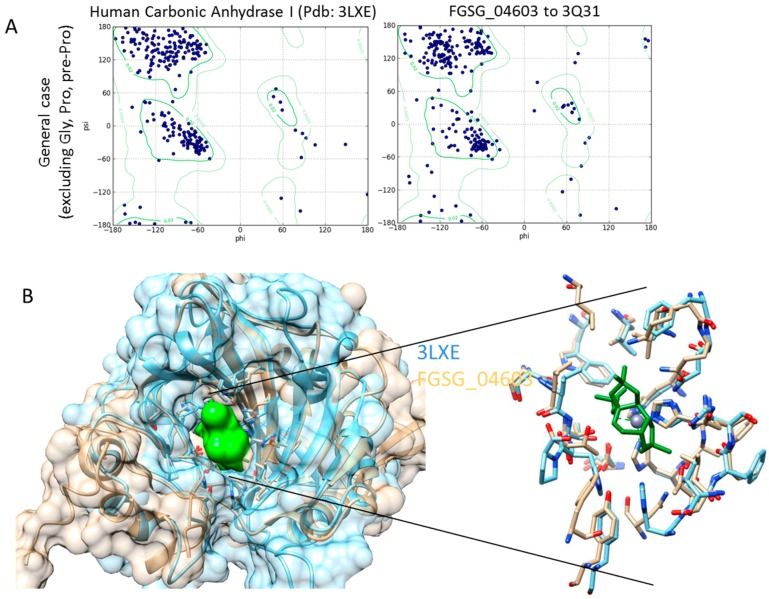
Model of *Fusarium graminearum* CA (FGSG_04603) in comparison to human CA. (**A**) Ramachadran plots showing protein backbone dihedral angles for amino acids. Dots present the angles (psi vs. phi) and outlined are energetically preferred angles. Shown are values for measured and calculated protein structures for human CA1 (measured) and fungus (calculated); and (**B**) comparison of structures of human and fungal CAI binding regions with inhibitory molecules included in green.

Figure 4A shows Ramachandran plots of the 3D structure of human carbonic anhydrase I (3LXE) and the homology model of protein FGSG_04603 is modeled using the 3Q31 crystal structure. Figure 4B shows a structural comparison between human carbonic anhydrase 1 (3LXE) and the model of *Fusarium graminearum* carbonic anhydrase FGSG_04603 in space filling and skeletal format. A skeletal format compares active sites of the carbonic anhydrase proteins following structure alignment. Additionally, the inhibitor (green), in order to mark the active site of the two proteins, is shown.

Structure quality has been assessed using Ramachandran plots for the model (Figure 4A). A comparison of Ramachandran plots for the 3LXE structure and the model show a similar distribution of residues in structural groups of the β-sheet, as well as the left- and right-handed α-helix. A large majority (over 90%) of residues (excluding glycine and proline, not shown here) fit within the allowed regions for both the published X-ray structure and our homology model. A visual inspection of structures and, particularly, residues within the active site, show similarities between 3LXE and modelled FGSG_04603 structures (Figure 4B). The majority of the residues in the vicinity of the active site are identical in the two proteins, suggesting similar activity, as well as inhibitory characteristics.

As only a relatively small number of RR metabolites have been experimentally tested, other possibly stronger inhibitors may exist within the set. In order to assist the selection of metabolites for further experimental analysis binding energy of RR metabolites have been tested computationally using high-throughput docking analysis performed using PyRx (Scripps Research Institute, La Jolla, CA, USA) [63] and AutoDock4 (Scripps Research Institute, La Jolla, CA, USA) [64]. A zinc atom has been added to the FGSG_04603 structural model according to the analysis of the structural overlap between the experimental and modeled data. Charges were first calculated using the Geisteiger model (under AutoDock4). Subsequently the zinc atom was assigned a charge of +2 and designated as not chemically bonded to the protein. Three-dimensional structures of all ligands were obtained from the PubChem database [38] and were used directly in PyRx. AutoDock4 analysis was performed for selected metabolites, including the assignment of charges to ligands using the Geisteiger model. All single bonds (outside of rings) in ligands were rotatable while the protein was kept rigid. High-throughput docking analysis was performed for both human and *Fusarium graminearum* proteins. Several compounds were selected for further testing based on their binding energies that previously indicated, experimentally, relevance as antifungal agents, as well as having proven activity as inhibitors of proteins in mammalian cells that have close orthologes in *Fusarium graminearum*. Further testing of other proposed *Fusarium graminearum* CA inhibitors are underway in our group.

### 2.4. Experimental Analysis

**Figure 5:**
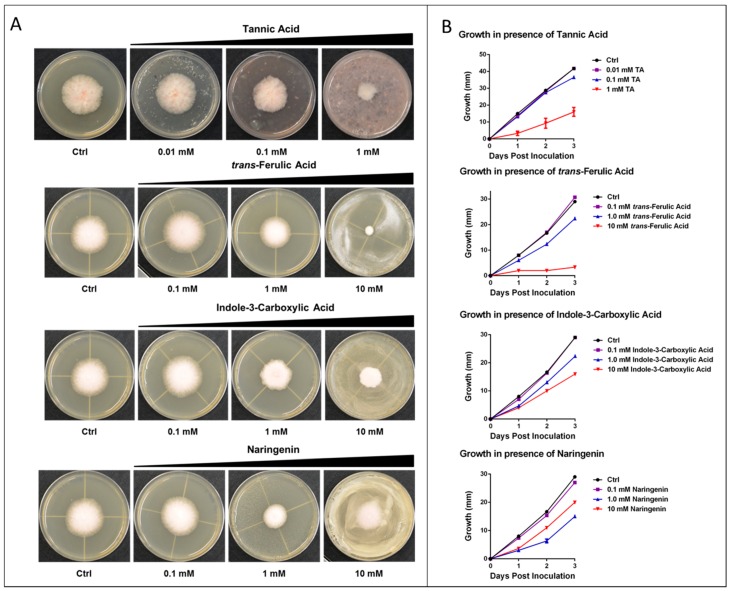
Fungal growth inhibition assay in the presence of increasing metabolite concentrations. (**A**) Radial growth of a fungal colony inoculated at the center of a potato dextrose agar (PDA) plate in the presence of different metabolites after three days post inoculation; and (**B**) radial fungal growth (diameter in mm) of a *Fusarium graminearum* inoculum measured over three days in the presence of increasing concentrations of each metabolite. Each point shows the average of three replicates ± standard deviation.

Several selected metabolites from the set of bibliome and computationally-determined binders to CA were tested against *Fusarium graminearum* in fungal growth inhibition assays, including tannic acid, trans-Ferulic acid, naringenin, and *N*-(*p*-coumaroyl) serotonin. The effect of these compounds on *Fusarium graminearum* growth in cultures is shown in Figure 5 with IC_50_ values listed in Table 1.

The strongest inhibitor of *Fusarium graminearum* growth of the tested metabolites is tannic acid, followed by naringenin and ferlic acid. These compounds have been shown previously to be highly promiscuous inhibitors of a range of metalloenzymes in mammalian cells including CA, P450, and lipoxygenase. The majority of protein targets for tannic and ferulic acid have close sequence orthologs in *Fusarium graminearum* and many (e.g., CA, P450, lipoxygenase) are important in *Fusarium graminearum* growth and virulence. Structural analysis of these proteins has shown significant similarity, particularly in the binding/active-site regions, suggesting activity of similar inhibitory molecules.

## 3. Materials and Methods

### 3.1. Metabolite Characterization

Metabolite information was obtained from automated and manual searching of all available literature dealing with the subject of metabolite or metabolomics analysis in wheat, barley, or oats infected with *Fusarium graminearum*. In addition, plant hormones, as well as metabolites listed as generally related to biotic responses are included. All metabolites determined from the literature have been mapped to the PubChem database [38] and CIDs were assigned. Molecular characteristics have been determined using ChemmineR [65], RCPI (developed by N. Xiao), and RCDK (developed by R. Guha), running under R and Bioconductor, and using 3D molecular structures for the analysis of physiochemical characteristics of molecules. These methods provided constitutional, topological, geometrical, and electronic descriptors, as well as molecular fingerprints, and were used to determine overall molecular characteristics, e.g., size; drug-likeness with Lipinski rule-of-five properties, and molecular chemical structure similarities. For a comparison of molecular characteristics we have also obtained all known plant metabolites from the Golm database [41], as well as all FDA approved drugs obtained from the ZINC database [44].

### 3.2. Protein Target Analysis

Experimentally-determined protein targets of metabolites have been obtained from the PubChem Database [38] using Bioactivity information. Only assays with known protein targets and with metabolites shown to be active have been retained. Several Perl and R scripts have been written in-house to search for protein information relating bioassay IDs with protein target IDs with protein names and structures. As most publicly available bioassay testing has been done for general resistance metabolites we have determined related metabolites in the selected group of resistance-related metabolites studied here using a structure similarity search provided under ChemmineR [65].

### 3.3. Sequence Comparison

*Homo sapiens* to *Fusarium graminearum* sequence comparisons have been performed using methods developed in-house. In this approach gene and protein sequences across species are compared in order to determine possible orthologs. Input sequences representing 13,321 protein encoding gene models for *Fusarium graminearum* were obtained from the Broad Institute Fusarium Comparative Database [56]. 89,033 Human protein sequences were acquired from the October 2014 release of the UniProt knowledge base [52].

The OrthoPred tool was applied to the input protein sequences using bidirectional BLAST local alignments (*e*-values greater or equal to 1 × 10^−4^ and word seed equal to 3) and detected 2930 one-to-one orthologs between the two species. The average BLAST e-values for *Fusarium*-to-human and human-to-*Fusarium* mappings were 5.5 × 10^−7^ and 1.9 × 10^−7^.

### 3.4. Homology Modelling

Three-dimensional structures of proteins for *Fusarium graminearum* have been obtained using homology modelling methods provided by Swiss-Model [60,61,62]. Swiss-Model is a web-based, fully-automated protein structure homology modelling tool. It first performs identification of one or more known protein structures with some sequence identity to the studied protein. Following users’ selection of homologous sequences for modeling, the software determines optimal 3D structures. Structures determined by Swiss-Model were validated using Ramachadran plot analysis (calculated using UCSF Chimera 1.9 (UCSF, San Francisco, CA, USA) and structure comparison with related proteins.

### 3.5. Docking Analysis

Docking was performed using AutoDock4 (Scripps Research Institute, La Jolla, CA, USA). AutoDock4 performs a rapid energy evaluation through a pre-calculated grid of affinity potentials. The macromolecule is kept rigid while torsional flexibility is allowed for the ligand molecule. The grid box for all calculations included active sites based on X-ray and literature information. Initial structures of all ligands were obtained from PubChem [38]. Following the addition of hydrogen atoms, all ligand structures were optimized using Gaussian 97 software using a semi-empirical optimization model. Atom charges in ligands were calculated using the Gasteiger method in Autodock4.

### 3.6. *Fusarium graminearum* Dose-Response Growth Assays

Tannic acid, *trans*-ferulic acid, indole-3-carboxylic acid and naringenin were purchased from Sigma-Aldrich. The effects of increasing concentrations of several metabolites on the hyphal growth of *Fusarium graminearum* were tested on potato dextrose agar (PDA) plates. Briefly, metabolites dissolved in various solvents (tannic acid in water, *trans*-ferulic acid in ethanol, indole-3-carboxylic acid in ethanol, and naringenin in ethanol) were spread on PDA plates (25 mL media per plate) to obtain a final metabolite concentration of 0.01–10 mM. Equal volumes of water or ethanol were spread onto control plates according to the solvent used to dissolve the various metabolites. The plates were incubated for an hour to let the metabolites absorb into the media. A small plug of *Fusarium graminearum* Z-3639 was placed at the center of each plate and the plates were incubated under constant light at 27 °C for three days. The diameter of fungal colonies in each plate was measured and recorded each day after inoculation.

## 4. Conclusions

Cheminformatics analysis has shown drug-like characteristics for RR metabolites observed in resistant grains infected with FHB. Some of the known protein binders for several RR metabolites had close homologs in the *Fusarium graminearum* proteome, suggesting possible targets for inhibition by RR metabolites. High-throughput computational docking analysis of interactions between RR metabolites and possible target proteins have shown that several of the RR metabolites can potentially act as significant inhibitors of carbonic anhydrase in *Fusarium graminearum*. Many of these metabolites result from secondary pathways stemming from tryptophan and include serotonin derivatives produced in the hydroxycinnamic acid and serotonin amide biosynthesis pathway, as well as products of IAA biosynthesis (auxin) and flavones, resulting from phenylalanine biosynthesis. Previously, experimental studies confirmed anti-fungal activity of several of these selected compounds, and herein we show that some have a significant effect on *Fusarium graminearum* hyphal growth. Further in planta tests investigating the ability of the compounds tested in vitro herein, as well as several other selected metabolites, to reduce FHB on wheat, are currently underway, aiming towards better understanding of possible antifungal targets, as well as focused modification of wheat to produce higher concentration of relevant RR metabolites.

## Figures and Tables

**Table 1:** IC_50_ values for tested compounds determined against *Fusarium graminearum* cell cultures.

Compound	IC_50_ (mM)
Tannic Acid	0.58
*trans*-Ferulic Acid	3.1
Naringenin	~ 1
indole-3-carboxylic acid	> 10

## Supplementary Materials

The following are available online at http://www.mdpi.com/2218-1989/6/4/31/s1, Table S1: List of resistance related metabolites obtained from referenced publications with their plant origin.
