# Supplementary material for: Metabolomics and Cheminformatics Analysis of Antifungal Function of Plant Metabolites
Source: Metabolites. 2016 Sep 30;6(4):31. doi: 10.3390/metabo6040031 (PMC5192437; doi:10.3390/metabo6040031)
Supplement: Supplementary file 1 [file metabolites-06-00031-s001.pdf]

Supplementary Materials: Metabolomics and Cheminformatics Analysis of Antifungal Function of Plant Metabolites

Miroslava Cuperlovic-Culf, NandhaKishore Rajagopalan, Dan Tulpan and Michele C. Loewen

List of resistance related metabolites obtained from referenced publications with their plant origin.

Pubchem (CID)	Metabolite	Synonim	Plant Origin	Reference
179	3-hydroxy-2-butanone	-	Chickpea volatiles	Cruz, 2012
445154	Resveratrol	-	General resistance	Lattanzio, 2006
199	Agmatine	-	Wheat	Gunnaiah, 2012
243	Benzoic acid	-	Wheat	Hamzehzarghani, 2005
264	Butanoic acid	-	Wheat	Hamzehzarghani, thesis
273	Cadaverine	1,5-Diaminopentane	Wheat	Hamzehzarghani, PhD
311	Citric acid	-	Barley	Bollina, 2010
323	Coumarin	-	barley	Chamarthi, 2014
338	*p*-hydroxybenzoic acid	salicylic acid	Wheat	Gunnaiah, 2012
370	5-*O*-β-glucoside of gentisic acid, gallic acid	gallic acid	Wheat	Boutigny, 2010
424	Aspartic acid	-	Wheat; Barley	Hamzehzarghani, PhD; Bollina, 2010
441	β-D-glucopyranosyl-sinapic acid	beta-Hydroxybutyric acid	Wheat	Gunnaiah, 2012
469	2-Aminoadipic acid	-	Barley	Bollina, 2010
674	dimethylamine	-	Chickpea volatiles	Cruz, 2012
750	Glycine	-	Wheat	Hamzehzarghani, PhD
753	Glycerol	-	barley	Bollina, 2011
754	glycerol-3-phosphate	-	signaling molecule	Dempsey, 2012
760	Glyoxylate/Oxaloacetiec acid	-	Wheat (Sumai-3)	Gunnaiah, 2014
801	auxin	-	hormon	Petti, 2012
802	Indole-3-acetate	-	wheat	Gunnaiah, PhD
811	2-methylenesuccinic acid	Itaconic acid	Wheat	Gunnaiah, PhD
847	Methionine sulfoxide	-	Barley	Bollina, 2011
849	pipecolic acid	-	Systemic acquired resistance factor	D'maris, 2012
867	Malonic acid	Malonic acid	Wheat; Barley	Hamzehzarghi, 2005; Bollina, 2010
892	Myoinositol	-	Wheat	Gunnaiah, PhD; Hamzehzarghani, 2012
931	naphthalene	-	Chickpea volatiles	Cruz, 2012
932	naringenin	-	Wheat; barley	Gunnaiah, PhD; Bollina, 2011

985	palmitic acid	Hexadecanoic acid	Wheat	Hamzehzarghi, 2005
991	Briaexcavatin O	Parathion	Barley	Bollina, 2011
999	benzeneacetic acid	-	wheat	Paranidharan, 2008
1004	Phosphoric acid	-	Wheat	Hamzehzarghi, 2005
1032	Propanoic acid	-	Wheat	Hamzehzarghi, 2005
1045	putrescine	1,4-Butanediamine	Wheat	Hamzehzarghi, PhD
1057	Pyrogallol	-	Barley	Bollina, 2011
1060	Pyruvic acid	-	Barley	Bollina, 2010
1103	spermine	-	Wheat	Gunnaiah, PhD
1110	Succinic Acid	Butanedioic acid	Wheat	Hamzehzarghi, PhD
1150	*tryptamine*	-	wheat	Pasquet, 2014
1662	3-hydroxy-3-methylglutaric acid	-	barley	Bollina, 2011
1826	3-Indoleacetic Acid	5-Hydroxyindoleacetic acid	wheat	Gunnaiah, PhD
2102	α-cyano-4-hydroxycinnamic acid	-	barley	Bollina, 2011
2266	azelaic acid	-	Systemic acquired resistance factor	D'Maris, 2012
2519	Caffeine	Guaranine	barley	Chamarthi, 2014
2969	Capric acid	-	Barley	Bollina, 2010
3301	1,2-Ethanediamine	-	Wheat	Hamzehzarghani, 2005
3314	Eugenol	-	Wheat (Sumai-3)	Gunnaiah, 2014
3449	(*S*)-Malate	-	Wheat (Sumai-3)	Gunnaiah, 2014
3830	cytokinin	-	plant hormon	Bari, 2009
3893	Lauric acid	-	Barley	Bollina, 2010
4133	methyl salicylate	-	Systemic acquired resistance factor	D'Maris, 2012
4992	*N*-Methylheteropsine	Pyrilamine	barley	Bollina, 2011
5192	Sebacic acid	-	wheat	Gunnaiah, PhD
5202	Serotonin	-	wheat	Gunnaiah, PhD; Pasquet, 2014
5281	Octadecanoic acid	stearic acid	Wheat	Hamzehzarghani, 2012
5950	L-Alanine	-	Wheat	Hamzehzarghani, PhD
5960	L-aspartate	-	wheat	Gunnaiah, PhD
5961	Glutamine	-	wheat	Hamzehzarghi, PhD
5984	D-Fructose	-	Wheat	Hamzehzarghani, 2005
5988	*O*-a-D-Glucopyranoside	Sucrose	Wheat	Hamzehzarghani, 2012
6036	galactose	-	Wheat	Hamzehzarghani, PhD; Paranidharan, 2008
6137	Methionine	-	barley	Bollina, 2011
6140	L-phenylalanine	-	Wheat	Gunnaiah, 2012
6251	D-Mannitol	-	Wheat	Gunniah, PhD
6262	ornithine	-	wheat	Paranidharan, 2008

6267	L-asparagine	-	wheat	Paranidharan, 2008; Gunnaiah, 2014
6274	Histidine	-	Wheat (Sumai-3)	Gunnaiah, 2014
6305	L-Tryptophan,	-	Wheat (Sumai-3)	Gunnaiah, 2014; Pasquet, 2014
6306	Isoleucine	-	barley	Bollina, 2011
6322	arginine	L-Arginine	barley	Bollina, 2010
6325	ethylene	-	plant hormon	Bari, 2009
6466	gibberellin	-	plant hormon	Bari, 2009
6508	Quinic acid	-	barley; wheat	Bollina, 2010; Gunniah, PhD
6560	2-methyl-1-propanol	-	Chickpea volatiles	Cruz, 2012
6581	2-Propenoic acid	acrylic acid	Wheat	Hamzehzarghani, PhD
6613	Pantothenic acid	-	barley	Bollina, 2011
6802	Guanosine	-	barley; Wheat (Sumai-3)	Bollina, 2011;Gunnaiah, 2014
7150	Methylbenzoate	-	Wheat	Gunniah, PhD
7311	2,4-*bis*(1,1-dimethylethyl)-, Phenol	2,4-di-tert-butylphenol	Wheat	Hamzehzarghani, 2012
7405	Pyroglutamic acid	-	Barley	Bollina, 2010; 2011
7427	Trehalose	-	Wheat	Hamzehzarghani, PhD
7456	3-methyl-phenol	-	Chickpea volatiles	Cruz, 2012
7469	Hydroxyacetophenone	-	Wheat	Gunniah, PhD
7478	anisic acid	-	wheat	Paranidharan, 2008
7670	*N*-ethyl-benzenamine	-	Chickpea volatiles	Cruz, 2012
7720	2-ethyl-1-hexanol	-	Chickpea volatiles	Cruz, 2012
7966	Cyclohexanol	-	Wheat	Hamzehzarghani, PhD
8020	dimethoxy methane	-	Chickpea volatiles	Cruz, 2012
8103	1-hexanol	-	Barley	Bollina, 2011
8180	Undecanoic acid	-	Barley	Bollina, 2010
8468	vanillic acid	-	wheat	Bourigny, 2008
8629	UDP-glucose	-	Wheat; barley	Gunniah, PhD; Bollina, 2011
8742	Shikimic acid	-	wheat	Stamova, 2008
8914	nonanol	-	Chickpea volatiles	Cruz, 2012
8955	3β-Hydroxycinnamolide	-	Barley	Bollina, 2010
9064	Catechin	-	general resistance	Lattanzio, 2006
9958	4-Methylcatechol	-	Wheat	Gunniah, PhD
10185	dihydroquercetin	-	general resistance	Lattanzio, 2006
10286	4H-1-Benzopyran-4-one	-	Wheat	Hamzehzarghani, PhD
10465	Heptadecanoic acid	-	wheat; barley	Hamzehzarghani, 2005; Bollina 2011

10467	Arachidic acid	-	Barley; Wheat	Bollina, 2011; Paranidharan, 2008
10607	(−)-Podophyllotoxin	-	Wheat (Sumai-3)	Gunnaiah, 2014
10690	D-Gluconate	-	Barley	Bollina, 2010
10742	syringic acid	-	wheat	Bourigny, 2008
10932	beta-aminobutyric acid	-	hormon	Petti, 2012
11005	Myristic acid	Tetradecanoic acid	wheat	Hamzehzarghani, 2005
12020	1-penten-3-ol	-	Chickpea volatiles	Cruz, 2012
12530	Tridecanoic acid	-	wheat	Paranidharan, 2008
13577	trans-2-hexen-1-ol	-	Chickpea volatiles	Cruz, 2012
14259	Eicosanoic acid, methyl ester	-	wheat	Hamzehzarghani, 2005
14925	1,2,3-Propanetricarboxylic acid	tricarballylic acid	Wheat	Hamzehzarghani, 2012
15608	Tridecanoic acid, 12-methyl-, methyl ester	-	wheat	Hamzehzarghani, 2005
16251	3,4-dimethoxy-phenol	-	Chickpea volatiles	Cruz, 2012
28557	1,4,7,10,13,16-hexaoxacyclooctadecane	-	Chickpea volatiles	Cruz, 2012
31251	nonanoic acid-ethyl ester	-	Chickpea volatiles	Cruz, 2012
31260	3-methyl-1-butanol	-	Chickpea volatiles	Cruz, 2012
31404	butylated hydroxytoluene	-	Chickpea volatiles	Cruz, 2012
33037	glucaric acid	-	wheat	Paranidharan, 2008
34756	*S*-adenosyl-L-methionine	-	Wheat	Gunnaiah, 2012
47945	Dihydro-7-hydroxymyoporone	-	barley	Bollina, 2011
65084	Gallocatechin-4 β-ol	-	barley	Bollina, 2011
65098	norvaline	-	wheat	Paranidharan, 2008
68077	Tangeretin	-	barley	Bollina, 2011
68482	*N*-Benzoylanthranilic acid	-	barley	Bollina, 2011
69867	Indole-3-carboxylic acid β-d-glucopyranosyl ester	-	barley	Bollina, 2011
70639	3-Methylxanthine	-	barley	Bollina, 2010
72276	Epicatechin	-	barley	Bollina, 2011
72277	Epigallocatechin	-	Wheat (Sumai-3)	Gunnaiah, 2014
72965	Ailanthone	-	Wheat (Sumai-3)	Gunnaiah, 2014
73399	(+)-pinoresinol	-	Wheat (Sumai-3)	Gunnaiah, 2014
73432	Brusatol	-	barley	Chamarthi, 2014
75891	*N*-methyl-β-alanine	3-(methylamino)propanoic acid	Wheat (Sumai-3)	Gunnaiah, 2014
79025	D-Glucose	Glucopyranose	wheat	Hamzehzarghani, 2012
79075	2-Monostearin	-	wheat	Hamzehzarghani, 2012
83064	Ribofuranose-1,2,3,5-tetra	-	wheat	Hamzehzarghani, 2012
84298	Asperuloside	-	barley	Chamarthi, 2014
87691	Loganin	-	wheat	Gunnaiah, 2012
88708	Gentiopicrin	-	barley	Chamarthi, 2014
91458	Aucubin	-	wheat; Wheat (Sumai-3)	Gunnaiah, 2012; Gunnaiah, 2014

91493	6-Phospho-D-gluconate		wheat	Gunnaiah, PhD
92794	Prunin	Naringenin 7-*O*-beta-D-glucoside	barley; wheat	Chamarthi, 2014; Gunnaiah, 2012; Bollina, 2010
92817	Melezitose	-	Wheat	Hamzehzarghani, PhD
92836	2-Hydroxypalmitic acid	2-hydroxyhexadecanoic acid	Wheat	Gunnaiah, PhD
92891	L-Albizziine	-	Wheat (Sumai-3)	Gunnaiah, 2014
92904	Indolelactate	-	Wheat (Sumai-3)	Gunnaiah, 2014
94253	Juanislamin	sesquiterpenic lactone similar to:judaicin	barley	Bollina, 2010
95259	xylose	-	wheat	Hamzehzarghani, PhD
96118	4′,5,6,7-Tetramethoxyflavone	-	Wheat (Sumai-3)	Gunnaiah, 2014
97214	3′,5-Dihydroxy-4′,6,7-trimethoxyflavone	-	wheat	Gunnaiah, PhD
97332	Quercetin pentamethyl ether	-	barley	Bollina, 2011
99535	Podolide/Gibberlin A7	-	Wheat (Sumai-3)	Gunnaiah, 2014
99748	Phenylacetaldoxime	-	barley	Bollina, 2011
119205	Matairesinol	-	barley	Bollina, 2011
120678	Quinovic acid	-	barley	Bollina, 2010
121947	shikimate-3-phosphate	shikimic acid-3-phosphate	Wheat	Gunnaiah, PhD
122667	4′-Demethylpodophyllotoxin	-	barley	Bollina, 2011
124021	β-Glucogallin	-	barley	Bollina, 2010
128708	should be: 2-naphthol 6′-*O*-malonylglucoside	available id: 4-phenyl-6-*O*-malonylglucoside	Wheat (Sumai-3)	Gunnaiah, 2014
131248	Benzyl alcohol beta-D-xylopyranosyl (1->6)-beta-D-glucopyranoside	benzyl alcohol xylopyranosyl-(1-6)-glucopyranoside	Wheat	Gunnaiah, PhD
131420	Mucronulatol-(4->) naringenin	similar to: astraisoflavanin	barley	Bollina, 2007
145689	2-Hydroxy-3-methylcarbazole	-	barley	Bollina, 2011
145742	L-Proline	-	wheat	Hamzehzarghi, 2005
150893	Heptamethoxyflavone	-	wheat	Gunnaiah, PhD
151152	Trihydroxybutyric acid	-	wheat	Hamzehzarghi, PhD
152430	5,2′,5′-Trihydroxy-3,6,7,4′-tetramethoxyflavone 5′-glucoside	-	barley	Bollina, 2010
155094	6-Prenylnaringenin	-	barley; wheat	Bollina, 2010; Gunnaiah, PhD
158280	7,4′-dihydroxyflavan	-	barley	Bollina, 2011
159171	PM-toxin B	-	barley	Chamarthi, 2014
159223	PM-toxin A	-	barley	Chamarthi, 2014
159578	2-Oxo-6-dehydroxyneoanisatin	-	barley	Bollina, 2011
161496	Bruceine B	-	Wheat (Sumai-3)	Gunnaiah, 2014
165275	Mevalonic acid 5-pyrophosphate	5-Diphosphomevalonic acid	barley	Bollina, 2011
167792	gulose	-	wheat	Paranidharan, 2008
171489	5-*O*-methylembelin	-	barley	Chamarthi, 2014

173651	Compactin diol lactone	-	barley	Chamarthi, 2014
182279	Tarennoside	-	Wheat (Sumai-3)	Gunnaiah, 2014
193042	(−)-Dihydrocubebin	-	barley	Bollina, 2010
219899	Arabinoic acid	-	wheat	Hamzehzarghani, PhD
255320	Methyl pentofuranoside	Lyxofuranoside (similar)	Wheat	Hamzehzarghani, PhD
315709	3,6,7,4′-tetramethoxyflavone	-	barley	Bollina, 2007
332427	Lariciresinol	-	Wheat	Gunnaiah, PhD
345501	Deoxypodophyllotoxin	-	Wheat	Gunnaiah, 2012
354446	Loganin	-	Wheat (Sumai-3)	Gunnaiah, 2014
439155	2-*S*-adenosyl-L-homocysteine	-	Wheat	Gunnaiah, 2012
439230	Mevalonic acid	-	barley	Bollina, 2007
439503	Salicylic acid 2-*O*-b-D-glucoside	salicin	Wheat	Gunnaiah, 2012
439514	scopolin	Scopoloside	Wheat	Hamzehzarghani, PhD
439664	Chlorophyllide	-	Wheat (Sumai-3)	Gunnaiah, 2014
439709	β-D-fructofuranose/α-D-glucose	-	Wheat (Sumai-3)	Gunnaiah, 2014
440074	*trans-Hydroxy-D-proline*	-	Wheat	Gunnaiah, PhD
440349	*N*-Succinyl-L-amino-6-oxopimelate	-	Wheat (Sumai-3)	Gunnaiah, 2014
441802	Isobrucein A	-	barley; Wheat (Sumai-3)	Chamarthi, 2014; Gunnaiah, 2014
441905	Astragaloside III	-	barley	Bollina, 2010
442036	Hallactone B	-	barley; wheat	Chamarthi, 2014; Gunnaiah, PhD
442044	Inumakilactone A glycoside	-	barley	Chamarthi, 2014
442425	Ipolamiide	-	barley	Chamarthi, 2014
442428	2-Hydroxyisoflavanone naringenin	-	Wheat	Gunnaiah, 2012
442457	5′-Prenylhomoeriodictyol	-	Wheat (Sumai-3)	Gunnaiah, 2014
442670	Cajanol/flavonoids((+)-Pisatin)	-	Wheat (Sumai-3)	Gunnaiah, 2014
442770	(±)-5-deoxykievitone	-	barley	Bollina, 2011
442830	Acanthoside D	(-)-Syringaresinol di-beta-D-glucoside	barley	Chamarthi, 2014; Bollina, 2011
442831	1-Acetoxypinoresinol	-	barley	Bollina, 2011
442833	Cleistanthin A	-	Wheat (Sumai-3)	Gunnaiah, 2014
442836	Medioresinol 4′-*O*-beta-D-glucopyranoside	-	Wheat	Gunnaiah, 2012
443012	Valeroidine	-	barley	Bollina, 2011
443015	Podorhizol beta-D-glucoside	-	barley	Chamarthi, 2014
443024	Acanthoside B	-	Wheat (Sumai-3)	Gunnaiah, 2014
443320	16-epivellosimine	-	barley, wheat	Chamarthi, 2014, Gunnaiah, 2012
443328	Lamioside	-	Wheat (Sumai-3)	Gunnaiah, 2014
443335	Iridotrial glucoside	-	Wheat	Gunnaiah, 2012
443340	10-Hydroxyloganin	-	Wheat	Gunnaiah, 2012

443349	7-dehydrologanin tetraacetate	-	barley	Chamarthi, 2014
443352	Deutzioside	-	Wheat	Gunnaiah, 2012
444212	aconitic acid	-	wheat	Paranidharan, 2008
444539	cinnamic acid	-	Wheat	Gunnaiah, 2012; Hamazehzarghani, 2005
444791	D-Glucuronic acid	-	Wheat	Hamazehzarghani, PhD
444972	fumaric acid	-	wheat	Paranidharan, 2008
445638	Palmitoleate	-	barley	Chamarthi, 2014
445639	Oleic acid	-	barley	Bollina, 2011
445858	*cis*-ferulic acid	4-methoxycinnamic acid	wheat, barley	Boutigny, 2010; Bollina, 2011
452855	Trachelogenin/Medioresinol	-	Wheat	Gunnaiah, PhD
467296	Epicatechin 3-*O*-(3-*O*-methylgallate)	-	Wheat (Sumai-3)	Gunnaiah, 2014
514181	Phyllanthusmin B	-	Wheat	Gunnaiah, 2012
519748	Benzene, (1-Butylpentyl)-	-	Wheat	Hamazehzarghani, 2005
615196	1-DL-(indole-3-acetyl)-myo-inositol	-	barley	Bollina, 2011
621824	2,4,6-Tri-*t*-butylbenzenethiol	-	wheat	Hamzehzarghani, PhD
636543	Cadabicine	-	Wheat (Sumai-3)	Gunnaiah, 2014
636550	Formosanatin A	-	Wheat (Sumai-3)	Gunnaiah, 2014
637090	trans-2-Butenoic acid	crotonic acid	Wheat	Hamzehzarghani, PhD
637542	*p*-coumaric acid	-	wheat; barley	Boutigny, 2010; Hamazehzarghani, PhD; Bollina, 2010
637760	chalcones	-	general resistance	Lattanzio, 2006
637775	Sinapinic acid	SINAPIC ACID	Wheat; barley	Gunnaiah, 2012; Boutigny, 2010; Bollina, 2011
641663	trans-p-Ferulyl alcohol 4-O-[6-(2-methyl-3-hydroxypropionyl)] glucopyranoside	-	barley	Bollina,2010; 2011
643757	cis-aconitate	-	wheat	Gunnaiah, PhD
689043	caffeic acid	Caffeyl alcohol	Wheat	Gunnaiah, PhD
709625	Cinnamoylglycine	-	barley	Bollina, 2011
1560034	L-quinate	-	Wheat (Sumai-3)	Gunnaiah, 2014
3010930	Hydroxypinoresinol	-	Wheat (Sumai-3)	Gunnaiah, 2014
3035544	6-methoxypodophyllotoxin	-	barley	Chamarthi,2014
3036184	(3-Phenylpropionyl) glycine methyl ester	-	barley	Bollina, 2011
3084296	Citrusin B	-	Wheat (Sumai-3)	Gunnaiah, 2014
3084507	5,4′-dihydroxy-3,6,7,8,2′-pentamethoxyflavone	-	barley	Bollina, 2011
5273569	fraxetin	7,8-Dihydroxy-6-methoxycoumarin	barley	Bollina, 2011
5274622	threo-carolignan E	-	Wheat (Sumai-3)	Gunnaiah, 2014
5280372	Coniferin	-	Wheat; Wheat (Sumai-3)	Gunnaiah, 2012;

				Gunnaiah, 2014
5280378	Formononetin	-	Wheat	Gunnaiah, PhD
5280406	1-*O*-sinapoyl-beta-D-glucose	Trans-*p*-sinapoyl β-D-glucopyranoside	barley	Chamarthi, 2014; Bollina, 2011
5280443	Apigenin	-	barley	Bollina, 2011
5280445	Luteolin	-	Wheat	Gunnaiah, PhD
5280486	Raucaffricine	-	barley	Chamarthi, 2014
5280507	Sinapyl-alcohol	-	Wheat (Sumai-3)	Gunnaiah, 2014
5280536	Coniferyl aldehyde	-	Wheat	Gunnaiah, PhD
5280536	Coniferaldehyde	-	Wheat (Sumai-3)	Gunnaiah, 2014
5280637	Cynaroside	-	Wheat	Gunnaiah, PhD
5280802	Sinapaldehyde	-	Wheat	Gunnaiah, 2012; Gunnaiah, 2014
5280847	4-Hydroxycinnamyl alcohol 4-D-glucoside	-	Wheat (Sumai-3)	Gunnaiah, 2014
5280863	Kaempferol	-	barley	Bollina, 2010
5280896	(*s*)-(+)-Abscisic acid	Abscisic acid	barley	Bollina, 2011
5280933	gamma-Linolenic Acid	9,12,15-Octadecatrienoic acid	Wheat; barley	Gunnaiah, 2012; Hamzehzarghani, PhD; Bollina, 2011
5280934	α-Linolenate	-	barley; wheat	Chamarthi, 2014; Gunnaiah, 2014
5281166	jasmonic acid	-	Wheat	Gunnaiah, 2012
5281167	*cis*-3-hexen-1-ol	-	Chickpea volatiles	Cruz, 2012
5281168	trans-2-hexenal	-	Chickpea volatiles	Cruz, 2012
5281204	Tuberonic acid glucoside	-	barley	Chamarthi, 2014
5281380	Ceceline	-	Wheat (Sumai-3)	Gunnaiah, 2014
5281440	Eleganin	-	Wheat (Sumai-3)	Gunnaiah, 2014
5281542	Harpagoside	-	Wheat (Sumai-3)	Gunnaiah, 2014
5281601	5,6-Dimethoxyflavone	-	Wheat	Gunnaiah, 2012
5281638	6-Hydroxykaempferol	-	barley; wheat	Bollina, 2010; Gunnaiah, PhD
5281677	Quercetin 7,3′,4′-trimethyl ether	-	Wheat (Sumai-3)	Gunnaiah, 2014
5281691	Rhamnetin	-	Wheat (Sumai-3)	Gunnaiah, 2014
5281712	Astringin	-	barley	Bollina, 2011
5281724	4′-Prenyloxyresveratrol	-	barley; Wheat (Sumai-3)	Bollina, 2011; Gunnaiah, 2014
5281737	Macrophylline	-	barley	Bollina, 2011
5281760	1-Caffeoyl-4-deoxyquinic acid	-	Wheat	Gunnaiah, PhD
5281762	5-*O*-Caffeoylshikimic acid	-	Wheat	Gunnaiah, PhD
5281770	Diferulic acid	-	Wheat (Sumai-3)	Gunnaiah, 2014
5281789	Licoisoflavone A	-	barley	Bollina, 2010

5281796	Feruloylputrescine	-	Wheat	Gunnaiah, 2012
5281862	Urushiol III	-	barley	Chamarthi, 2014
5281929	(−)-jasmonic acid methyl ester	-	barley	Chamarthi, 2014
5282066	1,3-dihydroxy-*N*-methylacridone	-	barley	Chamarthi, 2014
5282224	Abscisic aldehyde	-	Wheat	Gunnaiah, 2012
5282225	Abscisic alcohol	-	Wheat	Gunnaiah, 2012
5282316	8,11-Octadecadienoic acid	-	wheat	Hamzehzarghani, PhD
5282745	9-Hexadecenoic acid	palmitoleic acid	Wheat	Hamzehzarghani, PhD
5282797	9,12-Octadecadienoic acid	-	barley	Bollina, 2011
5283028	Traumatic acid	-	barley	Chamarthi, 2014
5288227	Maleate	maleic acid	Wheat (Sumai-3)	Gunnaiah, 2014
5312942	8-oxo-9,11-octadecadiynoic acid	9,11-Octadecadiynoic acid, 8-oxo-	barley	Bollina, 2011
5314312	Methylcinnamate	-	Wheat	Gunnaiah, PhD
5315175	Vellosimine	-	Wheat (Sumai-3)	Gunnaiah, 2014
5315908	(+)-pinoresinol 4-*O*-(6-*O*-galloyl)-β-D-glucopyranoside	-	Wheat (Sumai-3)	Gunnaiah, 2014
5315911	Cinnamoyltyramine	-	Wheat	Gunnaiah, 2012
5316673	Kaempferol 3-rhamnoside	afzelin	wheat; barley	Gunnaiah, 2012; Bollina, 2010
5316860	Syringin	Syringoside	Wheat	Gunnaiah, 2012; Gunnaiah, 2014
5318045	(*Z*)-3-Hexenyl beta-d-glucopyranoside	AC1NSWB1	barley	Bollina, 2011
5318865	Kukoamine A	-	barley	Chamarthi, 2014
5320863	Quercetin-3-D-xyloside	Kaempferol 3-xyloside	barley	Bollina, 2011
5358847	Auriculatin	-	barley	Bollina, 2010
5366244	3,7,11,15-Tetramethyl-2-hexadecen-1-ol	-	Wheat	Hamzehzarghani, PhD
5379033	Dehydrodiisoeugenol	-	Wheat	Gunnaiah, PhD
5379081	5,6-Dihydroxy-7,8,4′-trimethoxyflavone	-	Wheat	Gunnaiah, PhD
5383409	Scoparin	-	barley	Bollina, 2010
5458272	Eupacunolin	-	barley	Chamarthi, 2014
5458468	(2*S*,3*S*)-2-Hydroxytridecane-1,2,3-tricarboxylate	-	Wheat (Sumai-3)	Gunnaiah, 2014
5458879	p-coumaroylserotonin	-	barley; wheat	Chamarthi, 2014; Gunnaiah, 2012; Pasquet, 2014
5461017	arachidate	icosanoate	barley	Chamarthi, 2014
5462438	Psychotrine	-	barley	Bollina, 2011
5481665	Isorhamnetin 3-rutinoside-7-glucoside	-	barley	Bollina, 2010
5486199	Kaempferitrin	-	Wheat	Gunnaiah, PhD
5716902	3-oxo-2-(2-entenyl) cyclopentaneoctanoic acid	-	barley	Bollina, 2011
5771760	1-Hexadecanoyl-sn-glycero-3-phospho-(1′-myo-inositol)	1-(9Z-octadecenoyl)-sn-glycero-3-phospho-(1′-myo-inositol)	Wheat	Gunnaiah, PhD
5862476	Geranyl cinnamate	AG-G-80757	barley	Bollina, 2011

5945639	alpha,alpha'-Diethyl-4,4'-bis(2-propynyloxy)stilbene Vendors	-	Wheat	Gunnaiah, PhD
5969616	Feruloylserotonin	-	Wheat	Gunnaiah. 2012; Gunnaiah, 2014; Pasquet, 2014
6325261	3′-*O*-beta-Glucopyranosyl plumbagic acid methyl ester	AC1O3DCX	barley	Bollina, 2011
6437066	Salannin	-	barley	Chamarthi, 2014
6438621	Seselinol	-	barley	Bollina, 2010; 2011
6439562	p-Coumaroylputrescine	-	Wheat	Gunnaiah, 2012
6440550	4-Coumaroylcholine	2-[(E)-3-(4-hydroxyphenyl)prop-2-enoyl]oxyethyl-trimethylazanium	Wheat	Gunnaiah, PhD
6440783	3-*O*-caffeoylquinic acid	-	barley	Bollina, 2011
6441485	xanthoxin	-	Wheat	Gunnaiah, 2012
6454248	Doryanine	-	barley	Bollina, 2011
6479876	Apigenin 7-*O*-β-D-glucuronide	-	Wheat; barley	Gunnaiah. PhD; Bollina, 2011
6992086	L-Glutamine	-	Wheat (Sumai-3)	Gunnaiah, 2014
6992089	L-asparagine	-	Wheat (Sumai-3)	Gunnaiah, 2014
6995277	D-threonine or Homoserine	-	Wheat (Sumai-3)	Gunnaiah, 2014
9543641	6Z-Octene-2,4-diynoic acid	-	Wheat	Gunnaiah, PhD
9547268	(6*RS*)-22-oxo-23,24,25,26,27-pentanorvitamin D3 6,19-sulfur dioxide adduct/(6*RS*)-22-oxo-23,24,25,26,27-pentanorcholecalciferol 6,19-sulfur dioxide adduct	-	Wheat (Sumai-3)	Gunnaiah, 2014
9548810	Omega-Hydroxydodecanoic acid	-	barley	Bollina, 2010
9548881	2,3-Dinor-8-iso-prostaglandin-F2alpha	2,3-Dinor-8-iso PGF2alpha	barley	Chamarthi, 2014
9548882	2,3-Dinor-8-iso prostaglandin F1alpha	2,3-Dinor-8-iso PGF1alpha	barley	Chamarthi, 2014
9799386	5-*O*-Feruloylquinic acid	-	Wheat (Sumai-3)	Gunnaiah, 2014; Pasquet, 2014
9817274	Sappanone a	-	barley	Bollina, 2011
9843255	Quercetagetin 5,6,7,3′,4′-pentamethyl ether	-	Wheat	Gunnaiah, PhD
9995324	Cinnamoylserotonin	-	barley; wheat	Chamarthi, 2014; Gunnaiah, PhD
10038072	Cyathocaline	-	barley	Bollina, 2011
10131281	*N*-decanoyl-l-homoserine lactone	CHEMBL8799	barley	Bollina, 2011
10345235	Segetalin B	-	barley	Bollina, 2010
10382485	6-Prenylapigenin	-	barley	Bollina, 2011
10427023	Phytocassane B	-	barley	Bollina, 2011
10483858	Segetalin A	-	barley	Bollina, 2011
10685477	Enterolactone	-	Wheat	Gunnaiah, PhD
10794070	Isogambogic acid	-	barley	Bollina, 2011

10877291	Arabinofuranose	-	Wheat	Hamzehzarghani, PhD
11067541	Catechin 5,7,3′-trimethyl ether	-	Wheat (Sumai-3)	Gunnaiah, 2014
11561034	Trans-Zeatin riboside monophosphate	-	barley	Bollina, 2011
11694869	dehydroabietinal	-	Systemic acquired resistance factor	D'Maris, 2012
11824478	Dehydrodiconiferyl alcohol	-	barley	Bollina, 2011
11825308	1 β-(3-Hydroxy-4,5-dimethoxyphenyl)-*O*-glucopyranoside	-	barley	Bollina, 2011
11953806	Vomilenine	-	Wheat	Gunnaiah, 2012
12306850	Anisatin	-	Wheat (Sumai-3)	Gunnaiah, 2014
13916049	Ferulic acid 7-*O*-glucoside	NP-015589	Wheat	Gunnaiah, 2012
13963770	5-hydroxy-7,8-dimethoxyflavanone	-	Wheat	Gunnaiah, 2012
14162696	Quercetin 3,7-dimethyl ether	-	barley	Bollina, 2011
14162697	Quercetin 3,5,3′-trimethyl ether	-	Wheat	Gunnaiah, PhD
14237661	Lupinisoflavone G	-	Wheat (Sumai-3)	Gunnaiah, 2014
14583601	Ulexone B	-	Wheat (Sumai-3)	Gunnaiah, 2014
14825498	Ramontoside	-	Wheat (Sumai-3)	Gunnaiah, 2014
15071430	Murranimbine	-	barley	Bollina, 2010
15838234	Unanisoflavan	-	Wheat	Gunnaiah, PhD
15939882	12-Cytisineacetamide	-	barley	Bollina, 2011
15954045	Mg-Protoporphyrin	-	barley	Chamarthi, 2014
16061038	9*S*-hydroxy-10*E*,12*Z*-octadecadienoic acid (9(*S*)-HODE)	-	Wheat	Gunnaiah, 2012
16129778	tannic acid	-	general resistance	Lattanzio, 2006
16659826	Caffeoylserotonin	-	Wheat	Gunnaiah, 2012
21226108	Secologanin	-	barley	Chamarthi, 2014; Bollina, 2011
21270557	9-oxo-nonanoate	-	barley	Chamarthi, 2014
21574272	grandmarin	-	barley	Bollina, 2007
21676366	Catechin 7-*O*-apiofuranoside	-	barley	Bollina, 2011
22298557	*cis*-Reserverattrol-3,4-*o*-β-glucoside	-	Wheat (Sumai-3)	Gunnaiah, 2014
23259413	Methylophiopogonone B	-	Wheat (Sumai-3)	Gunnaiah, 2014
23724737	Cyanidin 3-*O*-glucoside	-	barley	Bollina, 2011
24779499	2-valeryl-sn-glycero-3-phosphocholine	-	Wheat	Gunnaiah, PhD
24779520	1-pentyl-sn-glycero-3-phosphocholine	-	Wheat	Gunnaiah, PhD
24892796	Glutathionylaminopropylcadaverine	-	Wheat	Gunnaiah, PhD
25200803	3-hydroxy-15-dihydrolubimin	-	barley; Wheat (Sumai-3)	Chamarthi, 2014; Gunnaiah, 2014
25201445	Secologanate	-	Wheat (Sumai-3)	Gunnaiah, 2014
25201518	10,16-Dihydroxyhexadecanoate	-	barley	Bollina,2010
25202084	*O*-phospho-L-tyrosine	-	Wheat (Sumai-3)	Gunnaiah, 2014
25202368	Isovitexin-7-*O*-glucosyl-2″*O*-rhamnoside	-	barley	Chamarthi, 2014
25202794	Kaempferol-3-rhamnoside	-	Wheat (Sumai-3)	Gunnaiah, 2014

25203018	Isoscoparin	-	barley	Bollina, 2011
25244083	3-oxo-2-(*cis*-2′-pentenyl)-cyclopentane-1-octanoate	OPC8	barley	Chamarthi, 2014
25244213	4-coumaroylquinate	-	Wheat	Gunnaiah, 2012; Pasquet, 2014
25244310	Dihydroconiferyl alcohol glucoside	-	barley, wheat	Chamarthi, 2014; Gunnaiah, 2012; Gunnaiah, 2014
25244544	Sinapaldehyde glucoside	-	Wheat	Gunnaiah, 2012
25244639	13(*S*)-hydroperoxy linolenic acid	hydroperoxylinolenic acid	barley; wheat	Bollina, 2011;Chamarthi, 2014; Gunnaiah, 2012
25244952	7-deoxyloganin	-	Wheat (Sumai-3)	Gunnaiah, 2014
25245116	4-coumaroylshikimate	-	Wheat	Gunnaiah, 2012
25245404	8′-hydroxyabscisate	-	Wheat	Gunnaiah, 2012
25245507	18-oxo-oleate	-	barley	Chamarthi, 2014
25245514	4-coumaroylagmatine	*cis*-*p*-Coumaroylagmatine	barley, wheat	Chamarthi, 2014; Gunnaiah, 2012; Bollina, 2011
25245732	9,10-epoxy-18-hydroxystearate	-	barley	Chamarthi, 2014
25245966	*N*-Caffeoylputrescine	-	Wheat (Sumai-3)	Gunnaiah, 2014
25246059	12-oxo-*cis*-10,15-phytodienoate	-	barley	Chamarthi, 2014
40467846	2-phospho-D-glycerate	-	Wheat (Sumai-3)	Gunnaiah, 2014
42607580	2′,4′,4-Trihydroxy-3′,3-dimethoxychalcone 4′-*O*-glucoside	LMPK12120180	Wheat	Gunnaiah, PhD
42607603	Chalconaringenin 2\′-rhamnosyl-(1->4)-xyloside	-	Wheat	Gunnaiah, PhD
42607604	Chalconaringenin 2′-rhamnosyl-(1->4)-xyloside	-	Wheat (Sumai-3)	Gunnaiah, 2014
42607625	3,4\′,6\′-Trihydroxy-4,2\′-dimethoxychalcone 4\′-*O*-rutinoside	-	Wheat (Sumai-3)	Gunnaiah, 2014
42607738	Furano[2″,3″:6,7]aurone	-	Wheat (Sumai-3)	Gunnaiah, 2014
42607881	5-O-Methylleridol	-	barley	Bollina, 2011
42607913	5,7,4\′-Trihydroxyflavanone 7-*O*-arabinosylglucoside	-	Wheat	Gunnaiah, PhD
42607916	5,7,4\′-Trihydroxyflavonone 4\′-*O*-xylosylglucoside	-	Wheat	Gunnaiah, PhD
42607925	Naringenin 7-O-(2″,6″-di-O-α-rhamnopyranosyl)-β-glucopyranoside	-	Wheat (Sumai-3)	Gunnaiah, 2014
42608018	4′-Hydroxy-5,7,2′-trimethoxyflavanone 4′-rhamnosyl-(1->6)-glucoside	-	Wheat (Sumai-3)	Gunnaiah, 2014
42608074	5-Hydroxy-7,4′-dimethoxy-6,8-di-C-prenylflavanone 5-O-galactoside	-	Wheat (Sumai-3)	Gunnaiah, 2014
42608082	Persiconin	-	Wheat	Gunnaiah, PhD
44123342	Jasmonoyl valine	-	Wheat	Gunnaiah, 2012
44229226	Dopaquinone	-	Wheat (Sumai-3)	Gunnaiah, 2014
44237241	2-(6′-methylthio)hexylmalate	-	Wheat	Gunnaiah, PhD
44256626	Pelargonidin 3-rutinoside-7-(6-(*p*-hydroxybenzoyl)glucoside)	-	Wheat	Gunnaiah, PhD

44256707	Cyanidin 3-[6-(6-*p*-hydroxybenzoylglucosyl)-2-xylosylgalactoside]	-	Wheat (Sumai-3)	Gunnaiah, 2014
44257034	Malvidin 3-*O*-glucoside	-	Wheat (Sumai-3)	Gunnaiah, 2014
44257078	Catechin 3-*O*-α-L-rhamnoside	-	barley	Bollina, 2007
44257132	Pneumatopterin A	-	Wheat (Sumai-3)	Gunnaiah, 2014
44257154	Catechin-4-ol 3-*O*-β-D-galactopyranoside	-	barley	Bollina, 2011
44257384	Dalpanin	Vitexin 2″-*O*-(E)-ferulate	barley	Chamarthi, 2014; Bollina, 2011
44257722	Hemsleyanoside	-	barley	Bollina, 2010
44257728	Isovitexin 2″-O-(6‴-feruloyl)glucoside	-	Wheat (Sumai-3)	Gunnaiah, 2014
44257742	Isovitexin-7-*O*-xyloside	-	barley	Chamarthi, 2014
44257823	Apigenin 7- rutinoside	-	Wheat (Sumai-3)	Gunnaiah, 2014
44257987	Isoorientin 4′-*O*-glucoside-2″-*O*-(E)-caffeate	-	Wheat (Sumai-3)	Gunnaiah, 2014
44258179	Isoscoparin 7-*O*-glucoside	-	barley	Bollina, 2011
44258273	Tricin 7-rutinoside	-	barley	Bollina, 2011
44258526	7-Methoxy-5,6:3\′,4\′-bis(methylenedioxy)flavone	-	Wheat	Gunnaiah, PhD
44258551	Skullcapflavone I 2′-(4′-E-cinnamoylglucoside)	-	barley	Bollina, 2011
44258567	Isoscutellarein 7-xyloside	-	barley	Bollina, 2010
44258812	Kaempferol-3-glucoside-7-rhamnoside	-	barley	Chamarthi, 2014; Bollina, 2010
44259085	Kaempferide 3-glucoside-7-rhamnoside	-	barley	Bollina, 2011
44259493	Syringetin 3-rutinoside	-	barley	Bollina, 2010
44259592	Rhamnetin 3-rhamninoside	-	barley	Bollina, 2010
44259659	Quercetin 3-*O*-methyl 7-*O*-galactoside	-	barley	Bollina, 2011
44259865	5,4\′-Dihydroxy-3,6,3\′-trimethoxy-7-prenyloxyflavone	-	Wheat	Gunnaiah, PhD
44567511	Kadsulignan	-	Wheat (Sumai-3)	Gunnaiah, 2014
44577222	Vanilloloside	-	Wheat	Gunnaiah, PhD
46173242	4-coumaroyl-3-hydroxyagmatine	-	Wheat (Sumai-3)	Gunnaiah, 2014
46173376	Feruloylagmatine	-	Wheat	Gunnaiah, 2012
46173811	(+)-Abscisyl β-D-glucopyranoside	abscisic acid-beta-D-glucopyranosyl ester	Wheat	Gunnaiah, 2012; Gunnaiah, 2014
46891830	1-(9E-octadecenoyl)-sn-glycero-3-phospho-(1′-sn-glycerol)	-	Wheat	Gunnaiah, PhD
49859717	indole-3-butyryl-glucose	-	Wheat (Sumai-3)	Gunnaiah, 2014
51399546	7-Deoxyloganate	-	Wheat	Gunnaiah, 2012
52940260	(+)-lariciresinol	-	Wheat (Sumai-3)	Gunnaiah, 2014
52940854	Cryptomeridiol	-	barley	Chamarthi, 2014
53326135	Magnaldehyde B	-	Wheat	Gunnaiah, PhD
53486401	Secoisolariciresinol di-*O*-glucoside	-	Wheat	Gunnaiah, 2012
53887649	Pentadecnoic acid, 14-methyl-, methyl ester, (270)	-	Wheat	Hamzehzarghi, 2005
54710960	Sinapate	-	barley	Bollina, 2010
54740347	primary fluorescent chlorophyll catabolite	-	barley	Chamarthi, 2014

54758681	(+)-7-iso-jasmonoyl-L-isoleucine	-	Wheat	Gunnaiah, 2012
56660526	Cineracipadesin F	-	barley	Bollina, 2011
56670862	7-Methoxygambogellic acid	-	barley	Bollina, 2011
56936277	Butyl 3-*O*-β-D-glucopyranosyl-butanoate	-	Wheat (Sumai-3)	Gunnaiah, 2014
57397163	Piloside B	-	barley	Bollina, 2011
71442684	(+)-Rangiformic acid	similar to: 1-methoxycarbonylheptadecane-1,1,2-tr icarboxylate	barley	Bollina, 2011
71752010	Triacetyl resveratrol	-	barley	Bollina, 2011
54685734	Dimoracin	-	Wheat (Sumai-3)	Gunnaiah, 2014
471912	β-D-glucopyranosyl-caffeic acid	-	Wheat	Gunnaiah, 2012
**KNApSAcK (C_ID)**	**Metabolite**	**Synonim**	**Plant origin**	**Reference**
C00010776	Sylvestroside III	-	barley	Bollina, 2010
C00027255	6′-*O*-α-D-Xylopyranosylalangiside	-	barley	Bollina, 2010
C00022795	16-Diacetoxy-7 α-hydroxy-18-malonyloxy-ent-cleroda-3-ene	-	barley	Bollina, 2010; 2011
C00010714	7-*O*-(4-methoxycinnamoyl) tecomoside	-	barley	Bollina, 2010; 2011
C00025375	Acetyllycoclavine	-	barley	Bollina, 2011
C00028282	Fumariflorine	-	barley	Bollina, 2011
C00036128	Isovaleroyloxylinalool	-	barley	Bollina, 2011
C00037495	Methyl 6-*O*-*p*-trans-coumaroyl-β-D-glucopyranoside	-	barley	Bollina, 2011
C00019437	Auriculatin 4′-*O*-Glucoside	7,6-(2′′′,2′′′-dimethyl-2 H-pyrano)-2′,5-dihydroxy-8-(3,3-dimeth ylallyl)-isoflavone 4′-*O*-beta-D-glucoside	barley	Bollina, 2011
		-		
C00036353	7-Oxomatairesinol	-	Wheat	Gunnaiah, PhD
C00007576	Indole-3-carboxylic acid β-D-glucopyranosyl ester	-	Wheat (Sumai-3)	Gunnaiah, 2014
C00000120	alpha-*N*-carbomethoxyacetyl-D-4-chlorotryptophan	-	Wheat (Sumai-3)	Gunnaiah, 2014
		-		
C00013945	b-D-fructosyl-a-D-(6-*O*-(E))-feruloylglucoside	-	Wheat (Sumai-3)	Gunnaiah, 2014
C00024026	6′-*O*-(*p*-Coumaroyl)-procumbide	-	Wheat (Sumai-3)	Gunnaiah, 2014
C00050612	(+)-Medioresinol di-*O*-β-glucopyranoside	-	Wheat (Sumai-3)	Gunnaiah, 2014
**HMDB (HMDB_ID)**	**Metabolite**	**Synonim**	**Plant Origin**	**Reference**
HMDB41579	3b,6a-Dihydroxy-alpha-ionol 9-[apiosyl-(1->6)-glucoside]	-	Wheat (Sumai-3)	Gunnaiah, 2014
HMDB41676	4-Methyl-(−)-epigallocatechin 7-glucuronide	-	Wheat (Sumai-3)	Gunnaiah, 2014
HMDB32743	Phloroacetophenone-[xylosyl-(1->6)-glucoside]	-	Wheat (Sumai-3)	Gunnaiah, 2014
HMDB36214	6-Feruloylglucose 2,3,4-trihydroxy-3-methylbutylglycoside	-	Wheat (Sumai-3)	Gunnaiah, 2014
**ChEBI (CHEBID)**	**Metabolite**	**Synonim**	**Plant Origin**	**Reference**
CHEBI:16099	(E)-Feruloyl-3-(arabinosylxylose), *cis*-*p*-Coumaric acid 4-[apiosyl-(1->2)-glucoside]	-	Wheat (Sumai-3)	Gunnaiah, 2014
	16-Diacetoxy-7 a-hydroxy-18-malonyloxyent-cleroda-3-ene	-	barley	Bollina, 2011
